# Populations Norms for “SLURP”—An iPad App for Quantification of Visuomotor Coordination Testing

**DOI:** 10.3389/fnins.2019.00711

**Published:** 2019-07-10

**Authors:** Barbara M. Junghans, Sieu K. Khuu

**Affiliations:** School of Optometry and Vision Science, University of New South Wales Sydney, Sydney, NSW, Australia

**Keywords:** eye-hand coordination, brain function, visually-driven behavior, behavior assessment, population norms

## Abstract

Currently the integrity of brain function that drives behavior is predominantly measured in terms of pure motor function, yet most human behavior is visually driven. A means of easily quantifying such visually-driven brain function for comparison against population norms is lacking. Analysis of eye-hand coordination (EHC) using a digital game-like situation with downloadable spatio-temporal details has potential for clinicians and researchers. A simplified protocol for the Lee-Ryan EHC (Slurp) Test app for iPad® has been developed to monitor EHC. The two subtests selected, each of six quickly completed items with appeal to all ages, were found equivalent in terms of total errors/time and sensitive to developmental and aging milestones known to affect EHC. The sensitivity of outcomes due to the type of stylus being used during testing was also explored. Populations norms on 221 participants aged 5 to 80^+^years are presented for each test item according to two commonly used stylus types. The Slurp app uses two-dimensional space and is suited to clinicians for pre/post-intervention testing and to researchers in psychological, medical, and educational domains who are interested in understanding brain function.

## Introduction

Human behavior is largely driven by visual information (Bisley, 2011), making visual function the most appropriate measure of brain function. However, developmental visuomotor milestones and most traumatic brain injury and neurological diseases are predominantly monitored in terms of motor functions that are at best gross to measure. By comparison, involvement of visual-drivers in the measurement of visuomotor responses can potentially provide a highly refined and sensitive measure of neural action planning and the functional integrity of both visual and motor systems at gross and fine levels. The development of a portable app to easily and objectively quantify eye-hand coordination (EHC) in terms of spatial and temporal coordinates on a series of visuomotor tasks of varied difficulty would make such visually-driven assessment amenable and have significant potential across a wide variety of domains: psychological, medical (particularly neurological and ophthalmic), educational.

Each day a multitude of our physical movements are defined by goal-orientated reaching and grasping in response to a visual stimulus. The neuronal pathways involved in these visual oculo-motor and manual motor systems utilize prediction-mediated feedback relationships which have been extensively investigated in primates (for a review see Goodale, 2011). What underpins how successfully a task is carried out is one's dynamic assessment of associated spatial and temporal errors, and how one factors in muscular control, proprioception, practice effects, motor coordination, and aspects of cognitive function such as visual attention, perception, and memory (Crawford et al., 2004). Choice of an object to reach, touch, grasp, or even avoid, assumes executive planning that has already engaged endogenous and exogenously activated neural networks (Corbetta and Shulman, 2002). Such dorsal stream networks involve control and direction of selective visual attention, as well as the interaction of long term and working memory with the ventral visual stream as needed for exogenous identification and grasping of an object (for example, is it stationary or moving?), plus a semantic understanding of the object's characteristics. Successful grasping requires experience with an object's resilience, density, weight and texture to conceptualize a future action, plus use of appropriate subcortical (superior colliculus and pretectal accommodation areas) and frontal field visual pathways to redirect eye movements to shift and direct attention. Engagement of parieto-frontal dorsal networks along with aspects of motor and somatosensory networks is required to plan these goal-directed shifts of attention to appropriately “weight” the grasp suited to an object's characteristics (for example, whether fine, slippery, easily breakable, heavy, or if moving).

Various methods of quantifying visuomotor responses by measuring eye and hand coordination have been reported, for example, the Grooved Pegboard (Merker and Podell, 2011), Purdue pegboard (Gardner and Broman, 1979), cup stacking tasks (Ruff, 1993; Merker and Podell, 2011), or finger point-and-touch (Gao et al., 2010). These are manually timed activities and require subjective observations, thus creating potential practitioner bias. Other drawbacks are that these tasks lack novelty, are highly repetitive and relatively non-engaging, especially for older individuals. Furthermore, gross arm movements during most of the tests may introduce a confounder into interpretation of “EHC” performance (Binsted et al., 2001). Digital apparatuses that provide more objective measures are now available, such as the Dynavision (Vesia et al., 2008) and the Kinematic Assessment Tool (Culmer et al., 2009). However, these particular digital methods are costly, time-consuming and for the Dynavision quite large, making them impractical for routine clinical assessment. On the other hand, the Lee-Ryan Eye Hand Coordination Test (Lee et al., 2014) (now known as “Slurp” because each test item is a cartoon-shaped 3-D rendered animal- or geometric-shaped straw emerging from a milkshake that draws the milkshake up out of the glass during tracing) uses an inexpensive iPad® app with either a rubber-tipped stylus or a “biro”-style Bluetooth stylus (e.g., iPencil®). The device is preferentially used flat on a desk, eliminating upper arm involvement during testing. Further, the flatness of the tablet device reduces the demand on stereopsis which, if impaired, in itself can reduce the success of visually guided movements (Webber et al., 2008; Suttle et al., 2011). Results using a 13-item pilot version of the app indicate high repeatability and that its tasks are highly engaging for both young and older participants (Lee et al., 2014). An additional advantage of the Slurp test is that all temporo-spatial data is downloadable making the test useful as a research tool.

It is well-known from the assessment of hand movements and control, that visuomotor performance is a function of age and subject to developmental stages (Voelcker-Rehage, 2008). However, a criticism of existing EHC tests, including the Slurp EHC Test, is that few population norms have been established (Tiffin and Asher, 1948; Gardner and Broman, 1979; Ruff, 1993; Klavora and Esposito, 2002; Wicks et al., 2015). Notably, pilot data on 83 participants using the full 20-item version of the Slurp test revealed that depending on age it can take up to 30 min to complete (Junghans BM, et al. IOVS 2017;58:ARVO E-Abstract 5427), which detracts from its routine implementation. As this data also showed that after just one practice item there is no order effect during presentation of the remaining 19 test items, potential existed to select particular items to create two smaller subtests that are objective, much quicker, clinically-useful and equivalent measures to facilitate comparison of developmental or aging milestones or pre/post therapeutic interventions. Areas that may warrant multiple assessment of eye-hand coordination with reference to population norms cover the ophthalmic condition of amblyopia (Suttle et al., 2011), neurological conditions such as stroke (Low et al., 2017), Parkinsons disease (Boisseau et al., 2002), and acquired brain injury (Gao et al., 2010) or the psychological and educational situations of developmental coordination disorder (Bieber et al., 2016), autism spectrum disorder (Anzulewicz et al., 2016), etc.

As a simpler test protocol is highly desirable for the Slurp test, the current study aimed to describe age-norms for visually normal persons without cognitive or neural impairment on two statistically equivalent subtests of the Slurp EHC Test, under conditions using two readily available but quite different types of stylus.

## Materials and Methods

The study was approved by the University of New South Wales (UNSW) Human Research Ethics Advisory (HREA) Panel D: Biomedical. All participants were provided with a Participant Information Statement, and gave signed and informed consent after the study was explained in accordance with the Declaration of Helsinki. In the case of children who participated, one parent similarly signed consent, whereas the children themselves gave verbal consent. Participants were recruited as a result of flyers circulated within the School of Optometry and Vision Science and at one private optometric practice. All participants were screened for 20/20 equivalent visual acuity at near plus the absence of any history of motor, visual or cognitive impairment. Participants sourced from the private optometric practice had previously been determined to also have no binocular vision abnormalities.

By inspection of existing data on 83 subjects who had completed the full version of the Slurp EHC Test, two subsets of six items each were chosen such that each contained two easy, two moderate and two harder traces and yielded similar total error and total time scores. Subtest “A” (the “Dragonfly subset”) was created and contains the Square, Snail, Whale, Elephant, Slurp and Dragonfly items, and Subtest “B” (the “Octopus subset”) the Triangle, Rabbit, Monster, Unicorn, Cat, and Octopus items. The study sample size accords with an estimate of size of at least 96 participants from the normal population assuming a 95% confidence level and a margin of error of 10%. This sample size is consistent with previous studies, and our own work which has shown sufficient power with this number of participants.

Testing was carried out under conditions similar to those existing during the pilot data capture that led to the selection of subsets A and B. An Apple iPad® [Apple Inc., Cupertino, CA, USA, iPad Pro® iOS 11.1 Model A1674; 9.7 inch] was loaded with the Slurp Test (https://itunes.apple.com/us/app/slurp/id1148830242?mt=8) and the investigator selected out the respective test items from the 20 available. After explanation of how testing would proceed, all participants first undertook one item referred to as the “Castle” as a demonstration of how the test would be conducted, and to absorb any potential learning effect (Lee et al., 2014). For participants undertaking both subsets, Tests A and B were presented in random order. Within each subset, items were also presented in randomized order. Participants were seated at 33 cm from the iPad® for children or 40 cm for adults and viewed binocularly whilst wearing their habitual spectacle or contact lens correction. No scaling for distance was undertaken. Either an iPencil® [Apple Inc., Cupertino, CA, USA, Bluetooth Model A1603] or a typical rubber-tipped stylus in common use (Targus Slim Stylus for Smartphone, Anaheim California, USA) was used for tracing. The Slurp software monitors the location of the stylus on the iPad in terms of its perpendicular distance from the path along the midpoint of the 5.0 mm wide straw. The software is currently set such that when the stylus deviates in excess of 3.5 mm, the commencement of an error is indicated in the data set and contributes to the count of errors. The warning sound option was activated to alert when a tracing error had been made. At the conclusion of tracing, the participant's data set was emailed from the iPad, thus providing the number of errors made and the time taken to complete for each trace.

Participants were split into age groups in two ways: three age groupings across all ages, nine age groupings across all ages (numbers of participants in each age group are indicated in Tables 1–3). Data was analyzed using SPSS (Statistical Package for the Social Sciences version 25, IBM Corporation, New York, USA). A comparison between error scores and time taken to complete Set A and Set B was made using analysis of variance (ANOVA) with consideration of main effects due to gender, type of stylus used and age. The raw data supporting the conclusions of this manuscript will be made available by the authors, without undue reservation, to any qualified researcher.

**Table 1:** Mean error scores and time taken for individual test items in subset A and subset B according to type of stylus used and age (three age groups).

	**iPencil stylus**									**Rubber-tipped stylus**								
	**5–12 years**			**13–50 years**			**51–90 years**			**5–12 years**			**13–50 years**			**51–90 years**		
	**Mean** **(lower-upper 95%CI)**			**Mean** **(lower-upper 95%CI)**			**Mean** **(lower-upper 95%CI)**			**Mean** **(lower-upper 95%CI)**			**Mean** **(lower-upper 95%CI)**			**Mean** **(lower-upper 95%CI)**		
	**Errors**	**Time**	***N***	**Errors**	**Time**	***N***	**Errors**	**Time**	***N***	**Errors**	**Time**	***N***	**Errors**	**Time**	***N***	**Errors**	**Time**	***N***
**SET A**																		
Square	1.5 (0.6–2.4)	9.4 (6.3–12.6)	14	0.8 (0.6–1.0)	7.5 (7.1–8.0)	87	1.2 (0.7–1.7)	11.2 (10.0–12.3)	36	2.9 (1.6–4.3)	13.2 (9.8–16.6)	15	1.8 (0.9–2.7)	9.3 (7.0–11.7)	14	2.2 (1.3–3.2)	16.5 (12.6–20.4)	21
Snail	2.1 (0.8–3.5)	12.7 (10.5–15.0)	14	1.1 (0.8–1.4)	11.3 (10.6–11.9)	89	1.5 (0.9–2.1)	15.2 (13.7–16.8)	36	4.9 (3.9–5.9)	18.9 (16.1–21.8)	15	2.4 (1.1–3.8)	13.3 (9.6–17.1)	14	2.3 (1.0–3.6)	24 (18.8–29.2)	21
Dragonfly	4.5 (1.6–7.4)	23.4 (19.1–27.7)	14	1.3 (1.0–1.6)	16 (15.1–16.9)	90	1.9 (1.2–2.6)	23.8 (21.0–26.6)	35	8.8 (5.9–11.7)	30.8 (23.6–38.0)	15	4.7 (1.0–8.4)	25.2 (17.6–32.7)	15	7.3 (4.6–10.0)	44.2 (32.4–56.1)	21
Whale	3.9 (2.1–5.6)	26.2 (21.5–31.0)	14	2.6 (2.0–3.1)	21 (19.9–22.2)	88	3 (1.9–4.2)	32.1 (28.4–35.7)	36	14 (9.5–18.5)	45.2 (34.7–55.8)	15	4.7 (2.0–7.9)	31.1 (22.1–40.1)	15	9.2 (5.8–12.7)	56.4 (40.1–72.7)	21
Elephant	5.2 (2.0–8.3)	29.9 (24.7–35.2)	13	2.7 (1.9–3.5)	22.6 (21.3–23.9)	89	3.3 (2.0–4.5)	33.2 (29.8–36.7)	35	8.7 (6.4–11.0)	41.3 (35.0–47.7)	15	4.3 (2.1–6.5)	27.9 (20.9–34.8)	15	10.3 (6.4–14.3)	54.9 (44.8–65.1)	21
Slurp	8.8 (5.4–12.2)	33.4 (27.5–39.4)	14	3.1 (2.5–3.6)	22.6 (21.4–23.8)	90	5.8 (4.1–7.5)	36.1 31.9–40.2)	36	14.5 (10.9–18.0)	43 (36.2–49.7)	15	6.4 (3.2–9.6)	32.7 (25.1–40.3)	15	13.1 (8.8–17.4)	64.6 (47.1–82.0)	21
**SET B**																		
Triangle	2.1 (1.0–3.1)	7.7 (6.1–9.4)	14	0.9 (0.7–1.2)	6 (5.6–6.5)	90	1.3 (0.6–2.0)	9.3 (7.8–10.8)	36	2.9 (2.0–3.9)	8.1 (6.9–9.4)	18	0.9 (0.3–1.6)	5.8 (4.8–6.9)	16	16 (0.5–4.4)	4.4 (10.8–21.3)	20
Rabbit	1.9 (0.5–3.4)	12 (10.3–13.7)	14	0.6 (0.3–0.8)	9.7 (9.1–10.2)	89	0.8 (0.4–1.3)	14.1 (12.5–15.8)	35	6.2 (4.3–8.2)	18.1 (13.2–23.1)	18	0.8 (0.4–1.2)	10.8 (9.6–12.0)	16	23.4 (1.4–4.2)	3.1 (17.3–29.4)	20
Monster	3.6 (1.7–5.5)	21.9 (18.5–25.3)	13	1.3 (1.0–1.6)	16.3 (15.4–17.2)	90	3 (1.4–4.7)	24.7 (21.4–28.1)	35	9.9 (7.3–12.5)	27.1 (25.1–29.0)	18	3.1 (1.8–4.3)	21.9 (18.2–25.6)	16	41.5 (2.3–5.4)	3.5 (31.1–51.7)	19
Unicorn	5.7 (2.5–8.9)	31.7 (25.4–37.9)	14	2.6 (1.9–3.3)	22.6 (21.4–23.8)	90	2.7 (2.0–3.5)	30.9 (28.5–33.4)	36	18.1 (12.3–23.9)	50 (34.9–65.0)	18	3.6 (1.8–5.5)	25.4 (20.5–30.3)	16	52.2 (3.0–7.6)	5.4 (40.0–64.4)	20
Octopus	6.7 (2.8–10.6)	35.9 (28.9–42.9)	14	2.2 (1.8–2.6)	27.5 (25.8–29.2)	88	3.3 (2.3–4.4)	38.4 (34.8–42.0)	36	15.9 (12.6–19.2)	53.4 (44.0–62.8)	18	4.9 (3.1–6.7)	34.1 (29.3–38.8)	16	83.7 (6.2–13.0)	7.8 (59.8–107.6)	20
Cat	6.9 (3.7–10.2)	30.9 (26.0–35.7)	14	3.2 (2.5–3.9)	23.5 (22.1–24.9)	90	4.7 (3.2–6.2)	33.3 (29.9–36.6)	36	15.3 (10.2–20.5)	39 (34.5–43.4)	18	4.2 (2.6–5.7)	28.1 (22.4–33.8)	16	56.5 (5.8–10.1)	4.8 (44.5–68.6)	20

**Table 2:** Mean error scores and time taken for individual test items in subset A and subset B when using a Bluetooth stylus grouped into nine age groups.

	**5–8 years**			**9–12 years**			**13–20 years**			**21–30 years**			**31–40 years**			**41–50 years**			**51–60 years**			**61–70 years**			**71–90 years**		
	**Mean** **(lower-upper 95%CI)**			**Mean** **(lower-upper 95%CI)**			**Mean** **(lower-upper 95%CI)**			**Mean** **(lower-upper 95%CI)**			**Mean** **(lower-upper 95%CI)**			**Mean** **(lower-upper 95%CI)**			**Mean** **(lower-upper 95%CI)**			**Mean** **(lower-upper 95%CI)**			**Mean** **(lower-upper 95%CI)**		
	**Errors**	**Time**	***N***	**Errors**	**Time**	***N***	**Errors**	**Time**	***N***	**Errors**	**Time**	***N***	**Errors**	**Time**	***N***	**Errors**	**Time**	***N***	**Errors**	**Time**	***N***	**Errors**	**Time**	***N***	**Errors**	**Time**	***N***
**SET A BLUETOOTH STYLUS**																											
Square	1.9 (0.4–3.4)	9.2 (7.2–11.1)	8	1 (0.3–1.7)	9.5 (2.3–16.7)	6	0.9 (0.5–1.3)	7 (6.2–8.0)	31	0.7 (0.4–1.0)	8.12 (7.5–8.8)	34	1.3 (0.6–1.9)	7.45 (6.2–8.8)	12	0.6 (0.2–1.0)	7 (5.3–8.8)	10	1.1 (0.3–1.9)	10.5 (9.2–11.7)	21	1.5 (0.5–2.5)	10.1 (8.8–11.3)	8	1 (0.4–1.6)	14.4 (10.7–18.1)	7
Snail	4 (1.7–6.5)	15.8 (13.4–18.3)	8	0.5 (0.0–1.2)	9 (7.8–10.2)	6	1.2 (0.7–1.7)	11.4 (10.2–12.5)	33	1 (0.5–1.5)	11.5 (10.6–12.3)	35	1.1 (0.2–1.9)	10.3 (8.8–11.7)	11	1.1 (0.2–2.0)	11.3 (9.3–13.3)	10	1.3 (0.6–2.0)	14 (12.7–15.2)	21	0.8 (0.1–1.4)	13.3 (12.1–14.5)	8	2.9 (1.0–4.7)	21.3 (16.1–26.4)	7
Dragonfly	8.1 (2.8–13.4)	27.9 (23.2–32.7)	8	1.5 (0.2–2.8)	16.4 (13.2–19.6)	6	1.5 (0.9–2.1)	15.6 (14.0–17.2)	33	1 (0.5–1.4)	15.9 (14.6–17.2)	35	2 (0.9–3.1)	16.9 (13.9–19.8)	12	1 (0.4–1.6)	16.9 (13.7–20.1)	10	1.6 (0.8–2.4)	21 (18.9–23.1)	20	1.9 (0.9–2.9)	22.7 (20.1–25.4)	8	2.9 (0.5–5.3)	33.1 (22.7–43.5)	7
Whale	7.4 (2.1–12.6)	33.5 (29.0–38.0)	8	1.8 (0.8–2.9)	18.2 (13.5–22.8)	6	2.8 (1.8–3.7)	20.56 (18.6–22.5)	32	2.2 (1.4–3.0)	21.1 (19.5–22.6)	34	1.9 (0.5–3.4)	19.2 (17.3–21.1)	12	4.1 (1.9–6.3)	25.3 (20.1–30.6)	10	3 (1.5–4.6)	30.1 (26.0–34.2)	21	2.6 (0.9–4.4)	30.2 (25.7–34.7)	8	3.4 (0.2–6.7)	40.2 (27.9–52.6)	7
Elephant	7.8 (3.1–12.4)	37.6 (34.2–40.9)	8	3.2 (0.0–6.3)	21.3 (17.2–25.5)	6	2.2 (1.4–3.0)	21.1 (19.2–23.1)	32	3.3 (1.5–5.0)	24.2 (21.9–26.4)	35	2 (0.6–3.4)	22 (19.8–24.2)	12	3 (1.1–4.9)	23.2 (19.0–27.4)	10	3.9 (1.9–5.8)	34.5 (29.2–39.8)	21	1.8 (0.1–3.4)	28.6 (24.5–32.7)	8	3.2 (1.1–5.3)	34.9 (30.0–39.8)	6
Slurp	11 (5.7–16.3)	40.4 (35.4–45.5)	8	5.8 (3.4–8.3)	23.8 (17.6–30.0)	6	3.1 (2.2–4.0)	22 (19.8–24.2)	33	2.9 (2.2–3.7)	22.6 (20.9–24.3)	35	4.3 (1.9–6.6)	21.8 (20.0–23.5)	12	2.1 (1.4–2.8)	25.9 (20.3–31.5)	10	4.4 (3.0–5.9)	32.2 (29.1–35.3)	21	7.3 (2.8–11.7)	32.6 (27.1–38.1)	8	8.2 (2.6–13.7)	51.6 (38.3–64.9)	7
**SET B BLUETOOTH STYLUS**																											
Triangle	3.3 (1.7–4.8)	9.5 (8.2–10.8)	8	1.3 (0.2–2.4)	5.3 (2.9–7.7)	6	0.8 (0.3–1.2)	5.9 (5.0–6.8)	33	0.8 (0.5–1.2)	6.2 (5.6–6.7)	35	1.1 (0.4–1.8)	5.4 (4.7–6.0)	12	1.4 (0.4–2.4)	6.5 (64.8–8.1)	10	1.1 (0.2–2.0)	8.01 (6.5–9.7)	21	2 (0.1–3.9)	9.9 (7.6–12.1)	8	1.1 (0.1–2.2)	12.1 (6.8–17.4)	7
Rabbit	4.1 (0.7–7.6)	15.2 (11.9–18.5)	8	2.7 (0.0–6.8)	9.6 (8.0–11.2)	6	0.6 (0.3–0.8)	9.6 (8.6–10.5)	33	0.5 (0.2–0.9)	9.9 (9.0–10.7)	34	0.8 (0.0–1.6)	9.1 (7.8–10.3)	12	0.5 (0.0–1.2)	9.9 (8.1–11.6)	10	0.8 (0.2–1.4)	13.5 (11.4–15.7)	21	0.9 (0.0–1.9)	13.1 (11.1–15.1)	7	0.9 (0.2–1.5)	16.9 (12.3–21.5)	7
Monster	5.3 (1.9–8.7)	24.8 (22.3–27.3)	7	3.2 (0.0–7.4)	17 (12.5–21.6)	6	1.5 (1.0–2.0)	16 (14.5–17.4)	33	1.1 (0.6–1.6)	16.3 (15.1–17.5)	35	1.7 (0.8–2.6)	15.8 (14.0–17.5)	12	1.1 (0.3–1.9)	18.6 (13.3–23.9)	10	3.7 (1.0–6.3)	23.3 (19.2–27.5)	21	2.3 (0.8–3.7)	22.5 (18.4–26.7)	8	1.8 (0.7–3.0)	32.5 (21.9–43.0)	6
Unicorn	9.5 (2.2–16.8)	40.2 (32.9–47.5)	8	6.8 (0.0–16.3)	21.9 (17.5–26.2)	6	2.8 (1.5–4.0)	21.8 (19.7–24.0)	33	2.7 (1.5–3.9)	23.4 (21.5–25.2)	35	2.3 (1.2–3.5)	21 (18.6–23.3)	12	2.4 (0.2–4.6)	24.7 (20.7–28.7)	10	1.9 (1.1–2.7)	28.3 (25.6–31.0)	21	3.6 (1.7–5.5)	30.1 (25.8–34.3)	8	4.1 (2.3–6.0)	39.9 (35.1–44.7)	7
Octopus	9.6 (3.4–15.8)	44.9 (38.1–51.7)	8	3.5 (0.6–6.4)	23.9 (19.6–28.3)	6	2.3 (1.7–3.0)	25.8 (23.4–28.2)	32	2.1 (1.5–2.7)	27 (24.8–29.1)	34	1.9 (0.7–3.1)	27.2 (23.9–30.5)	12	2.5 (0.7–4.3)	35.5 (26.6–44.4)	10	3.3 (1.9–4.7)	35.8 (31.4–40.2)	21	3.1 (0.7–5.6)	38.4 (33.5–43.3)	8	3.7 (1.2–6.3)	46.2 (35.6–56.8)	7
Cat	8.4 (3.7–13.0)	36.8 (31.8–41.9)	8	4.3 (0.8–7.9)	23.6 (18.5–28.7)	6	3.2 (2.3–4.1)	23.1 (20.7–25.5)	33	2.9 (1.9–4.0)	22.7 (21.0–24.3)	35	3.3 (1.9–4.7)	23.2 (19.9–26.6)	12	3.8 (0.1–7.5)	28.3 (22.1–34.5)	10	6 (3.7–8.2)	31.5 (27.4–35.6)	21	1.9 (0.7–3.1)	28.6 (23.4–33.7)	8	4.1 (1.3–7.0)	43.6 (37.7–49.5)	7

**Table 3:** Mean error scores and time taken for individual test items in subset A and subset B when using a rubber-tipped stylus grouped into nine age groups.

	**5–8 years**			**9–12 years**			**13–20 years**			**21–30 years**			**31–40 years**			**41–50 years**			**51–60 years**			**61–70 years**			**71–90 years**		
	**Mean** **(lower-upper 95%CI)**			**Mean** **(lower-upper 95%CI)**			**Mean** **(lower-upper 95%CI)**			**Mean** **(lower-upper 95%CI)**			**Mean** **(lower-upper 95%CI)**			**Mean** **(lower-upper 95%CI)**			**Mean** **(lower-upper 95%CI)**			**Mean** **(lower-upper 95%CI)**			**Mean** **(lower-upper 95%CI)**		
	**Errors**	**Time**	***N***	**Errors**	**Time**	***N***	**Errors**	**Time**	***N***	**Errors**	**Time**	***N***	**Errors**	**Time**	***N***	**Errors**	**Time**	***N***	**Errors**	**Time**	***N***	**Errors**	**Time**	***N***	**Errors**	**Time**	***N***
**SET A RUBBER-TIPPED STYLUS**																											
Square	3.5 (1.6–5.4)	14.7 (9.7–19.6)	10	2.1 (1.1–3.2)	9.6 (7.4–11.8)	6	2.7 (1.0–4.3)	7.9 (5.9–9.9)	6	0.5 (0.0–1.5)	8.5 (0.8 (16.2)	2	0.5 (0.0–1.5)	7 (6.9–7.0)	2	1.8 (0.5–3.0)	13 (6.8–19.2)	4	2 (0.6–3.4)	16.2 (7.4–24.9)	6	2.3 (0.8–3.7)	11.7 (8.0–15.5)	8	2.4 (0.3–4.6)	22.2 (15.9–28.5)	7
Snail	4.9 (3.6–6.2)	20 (15.8–24.1)	10	4.5 (2.8–6.2)	15 (12.6–17.4)	6	1.5 (0.7–2.3)	9.4 (7.4–11.4)	6	2.5 (0.0–5.4)	10.6 (10.2–11.0)	2	0 (0.0–0.0)	11.3 (1.9–20.6)	2	5 (1.7–8.3)	21.7 (13.8–29.5)	4	3.2 (0.4–5.9)	22.3 (9.8–34.8)	6	2 (0.0–4.6)	18.7 (13.7–23.7)	8	1.9 (0.5–3.2)	31.5 (23.1–39.9)	7
Whale	10.7 (7.3–14.1)	34.5 (24.9–44.2)	10	4.7 (1.3–8.0)	19.7 (15.6–23.9)	6	3.2 (0.4–5.9)	17.5 (13.5–21.4)	6	0.5 (0.0–1.5)	18.9 (9.7–28.1)	2	1 (0.2–1.0)	20.2 (10.0–30.4)	3	12 (0.8–23.3)	43.6 (26.4–60.8)	4	4.8 (0.9–8.7)	42.9 (13.1–72.6)	6	7.4 (4.0–10.7)	29.6 (19.7–39.5)	8	9.3 (2.8–15.7)	62.2 (45.2–79.1)	7
Elephant	16.4 (10.2–22.6)	48.6 (32.3–64.9)	10	8.5 (5.6–11.4)	31.9 (29.2–34.6)	6	3.7 (1.2–6.1)	22.1 (16.6–27.5)	6	0.5 (0.0–1.5)	20.6 (17.1–24.0)	2	1.3 (0.0–3.1)	27 (12.8–41.2)	3	10.8 (4.7–16.8)	53.1 (33.6–72.5)	4	7.8 (2.3–13.4)	48.7 (17.9–79.4)	6	9.9 (3.2–16.5)	41 (28.6–53.4)	8	9.7 (3.7–15.7)	80.6 (46.1–115.2)	7
Dragonfly	9.4 (6.5–12.3)	41.5 (31.1–52.0)	10	7.3 (4.2–10.4)	33.3 (25.7–40.9)	6	3.5 (0.4–6.6)	19.6 (15.9–23.4)	6	3 (1.0–5.0)	24.5 (21.6–27.4)	2	2.7 (0.9–4.4)	29 (17.7–40.3)	3	7.3 (0.7–13.8)	41 (21.6–60.5)	4	10.5 (3.4–17.8)	53.9 (29.7–78.0)	6	11.9 (4.3–19.5)	44.2 (33.3–55.1)	8	8.4 (2.3–14.6)	68.1 (51.9–84.4)	7
Slurp	15.8 (10.8–20.8)	43.7 (33.0–54.4)	10	10.7 (7.2–14.2)	33.9 (25.8–42.1)	6	5.2 (2.7–7.6)	24.3 (19.8–28.8)	6	1.5 (0.5–2.5)	24.3 (22.4–26.2)	2	4.3 (0.7–8.0)	34 (29.6–38.3)	3	12.3 (2.5–22.0)	48.6 (27.5–69.6)	4	15.2 (3.5–26.8)	61 (27.7–94.3)	6	12 (6.4–17.6)	43.9 (35.0–52.8)	8	12.6 (5.9–19.2)	91.3 (54.2–128.3)	7
**SET B RUBBER-TIPPED STYLUS**																											
Triangle	2.9 (2.0–3.8)	8.0) (6.3–9.8	10	3 (1.4–4.6)	8.0) (6.3–9.8	9	0.8 (0.0–1.9)	5.3 (3.7–6.8)	6	0 (0.0–0.0)	4.1 (3.5–5.7)	3	1.6 (0.3–2.9)	6.5 (4.3–8.7)	5	1 (0.0–3.0)	7.9 (5.4–10.3)	2	0.6 (0.0–1.4)	9.9 (4.1–15.7)	5	2.2 (0.0–3.5)	14.7 (6.7–22.6)	9	4.3 (0.2–8.4)	23.2 (12.9–33.6)	6
Rabbit	7.3 (5.0–9.6)	20.6 (12.1–29.0)	10	4.3 (1.3–7.4)	20.6 (12.1–29.0)	9	1 (0.5–1.5)	9.2 (7.7–10.6)	6	0.7 (0.0–2.0)	9.4 (9.2–9.6)	3	0.8 (0.0–1.8)	12.1 (11.0–13.2)	5	0.5 (0.0–1.5)	14.7 13.5–15.9)	2	3 (0.0–6.6)	19.5 (8.9–30.1)	5	1.8 (0.3–3.3)	18.6 (11.9–25.2)	9	4.2 (1.6–6.7)	33.8 (20.9–46.7)	6
Monster	11.5 (8.7–14.3)	27.7 (23.9–31.5)	10	8 (3.9–12.1)	27.7 (23.9–31.5)	9	3.2 (1.1–5.2)	21.4 (16.6–26.7)	6	2 (0.0–5.9)	14.1 (12.1–20.0)	3	2.8 (0.9–4.7)	21.2 (14.3–28.0)	5	5 (1.1–8.9)	33.2 (29.5–36.9)	2	4.8 (1.9–7.7)	39.9 (12.7–67.2)	5	3.8 (1.1–6.4)	33.6 (19.9–47.3)	8	3.2 (0.3–6.0)	53.3 (40.5–66.1)	6
Unicorn	19.9 (12.5–27.3)	46.6 (34.4–58.8)	10	15.7 (7.4–23.9)	46.6 (34.4–58.8)	9	1 (0.1–3.5)	20.5 (16.7–24.1)	6	2 (0.0–4.3)	18.6 (17.6–21.3)	3	5.6 (1.7–9.5)	28.2 (22.4−34.0)	5	6.5 (0.0–15.3)	42.4 (17.5–67.3)	2	5.8 (0.1–11.5)	45.6 (15.5–75.7)	6	5 (0.9–9.1)	43.3 (29.1–57.5)	9	5.3 (2.5–8.0)	72.2 (55.6–88.7)	6
Octopus	15.9 (12.0–19.8)	58.7 (41.2–76.1)	10	15.1 (9.7–20.6)	58.7 (41.2–76.1)	9	5.2 (2.6–7.7)	27.8 (24.7–30.1)	6	3 (0.0–6.4)	27.1 (24.7–31.3)	3	5.4 (0.8–10.0)	40 (31.8–48.0)	5	6 (2.1–9.9)	48.9 (42.7–55.0)	2	14.2 (6.5–21.9)	93.6 (16.6–170.7)	5	8.6 (3.2–13.9)	69.6 (39.2–100.0)	9	7.3 (2.8–11.9)	96.4 (70.9–122.0)	6
Cat	15.5 (10.9–20.1)	39.1 (30.1–48.1)	10	14.2 (4.8–23.7)	39.1 (30.1–48.1)	9	2.7 (1.2–4.2)	23.3 (16.2–29.9)	6	3.7 (0.8–6.5)	23.3 (22.0–25.6)	3	6.4 (2.5–10.3)	31.2 (17.1–45.3)	5	4 (4.0–4.0)	41.7 (39.2–44.2)	2	9.4 (4.1–14.7)	56.9 (19.7–94.1)	5	7.3 (4.1–10.6)	44.6 (33.2–56.0)	9	7.7 (4.5–10.9)	74.1 (59.4–88.8)	6

## Results

The 221 participants who completed either Set A or B or both ranged in age from 5 to 88 years, with 60.5% female. Given there was no significant gender difference found for either errors made or times taken on each of the items, data from males and females of the same age group were pooled. Only 6.4% of the participants were left-handed and were scattered across age groups and type of stylus used. Inspection of rank order of error scores and time taken put left-handers fully within the range of scores found for right-handers and therefore their results were pooled. The mean error scores and time taken for 192 participants across all ages to complete Subset A (Dragonfly), regardless of stylus used, are 21.4 ± 21.6 errors and 139.2 ± 77.1 s, respectively. The mean error scores and time taken for 195 participants across all ages to complete Subset B (Octopus), regardless of stylus used, are 21.0 ± 23.2 errors and 142.8 ± 76.61 s, respectively. Despite the varying complexity and length of each test item, the ratio of errors per unit time was found to not differ significantly between items.

**Figure 1:**
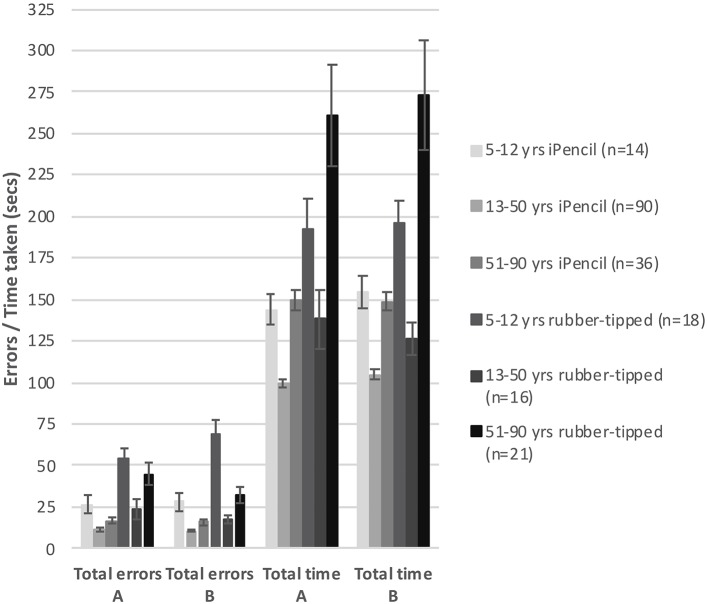
The bar graph shows mean total errors made and mean total time taken to complete subsets A and B against type of stylus used within three generalized age groups. Error bars represent 95% confidence intervals.

Two main factors relating to performance emerged; stylus and age. First, which type of stylus was used was found to be highly significant for both errors made (Subset A *F*_(1, 86)_ = 8.649, *p* = 0.004 and Subset B *F*_(1, 86)_ = 7.791, *p* = 0.006) and for time taken (Subset A *F*_(1, 86)_ = 29.552, *p* < 0.000 and Subset B *F*_(1, 86)_ = 28.908, *p* < 0.000). Of all participants, 73.3% completed Test A using an iPencil® and 26.7% used the rubber-tipped stylus, while Test B was completed by 72.2% using the iPencil®, and 27.8% the rubber-tipped stylus. Using a rubber-tipped stylus approximately doubles the number of errors, and increases the time taken by ~30% for those under 50 years and by 75% for those over 50 years of age (Figure 1 and Tables 1–3).

Second, age was also a significant factor in total errors made completing each subset (Figure 1) (Subset A *F*_(1, 86)_ = 2.563, *p* < 0.000 and Subset B *F*_(1, 86)_ = 2.596, *p* < 0.000) and the total time taken for each subset (Subset A *F*_(1, 86)_ = 17.627, *p* < 0.000 and Subset B *F*_(1, 86)_ = 15.230, *p* < 0.000), as well as in the errors made and time taken per individual test item (Tables 2, 3). With participants broken up into three age groups, analysis clearly indicated superior performance as regards both errors and time is achieved by those aged between 13 and 50 years (Figure 1 and Table 1). The consistency of this pattern can be seen in Figure 1, regardless of which subset of items is being undertaken or which stylus is used. Increasingly poorer performance was found as participants fell into the younger and older age groups moving away from the teenage/young adult years (Table 3). Additionally, tracing the most complexly shaped items amplified the poorer outcomes.

Set A and Set B were deemed comparable to each other with respect to total errors made/time taken, as the mean total errors made and the mean total time taken were not significantly different between Sets A and B, regardless whether a Bluetooth stylus or a rubber-tipped stylus was used (Figure 1). However, despite the mean total errors and time being similar for Sets A and B, the profile of increasing item complexity through the six items was not as similar as predicted. Set A appears to contain two easy items, two moderately complex items, and two complex items as predicted by pilot testing, whereas Set B has a less pronounced stepping of increasing complexity (two items are clearly easier, one item appears of moderate challenge and the remaining three items appear to be as hard as each other).

## Discussion

This study has demonstrated a quick, sensitive and objective means of assessing neural function in both pediatric and geriatric populations through analysis of the performance on an EHC task in an engaging two-dimensional space. EHC population norms across ages 5 to 80^+^ years have been determined for accuracy and timing on 12 variously complex tracing plates in the Slurp app. An age-related developmental effect with peak performance during young adulthood has been demonstrated. The automated timing/error-counting facility ensures subject and observer objectivity and thus addresses important issues against other forms of measuring visuomotor performance. The validation of Subtests A (Dragonfly) and B (Octopus) as statistically equivalent with respect to total errors made and total time taken for all age groups, indicates potential for reliable and valid pre-/post-intervention analysis of brain function. Each six-plate subtest can be completed in ~2–4 min, depending on age, making the app useful to clinicians in a range of disciplines such as neurology, optometry, psychology (including bedside assessment of patient visuomotor integrity), and education.

No gender difference was found using the Slurp EHC app. Consensus is poor regarding the influence gender has on visuomotor performance in three-dimensional space through assessment of skills based on throw and catch (Wickens, 1974; Plimpton and Regimbal, 1992; Wicks et al., 2015), reach and touch (Klavora and Esposito, 2002) or pick and place items (Ruff, 1993). Handedness may be considered as an issue. The relatively few left-handers yielded results dispersed along a similar spectrum as right-handers. It should be noted that each trace item requires a variety of leftwards/rightwards and upwards/downwards tracing, making certain sections of the test items problematic for both right and left-handers.

Importantly, a similar significant main effect for age has been measured by us in a two-dimensional space as by others in a three-dimensional that has implications for the study of developmental or aging effects of sensory-motor integration (Hay, 1979; Ruff, 1993; Smyth et al., 2004). In the current study, children under age 12 made a significantly greater number of errors and took significantly longer, demonstrating also a much greater variability in performance. It is known that children use vision in the control of hand movements in different ways according to age (Hay, 1979; Smyth et al., 2004) and therefore the greater variability and poorer EHC performance found in children in the current study, particularly under the age of nine, is not unexpected. “Attention and directing” studies reveal that younger children appear to employ visually-driven movements of the hand using a feedforward approach whereas by approximately age seven onwards they use a feedback-feedforward model that integrates proprioceptive information (Hay, 1979; Smyth et al., 2004), performance peaking in their teens. Developmentally, children continue to develop throw/catch visual coordination skills into their mid-teens, with boys outperforming girls (Wicks et al., 2015). The Purdue Pegboard Test has been used to establish “pick and place” normative data on children (*n* = 1,334, ages 5–16 years) (Gardner and Broman, 1979) and indicated improved speed until approximately age 10 years, after which performance leveled.

The impact of a poor functional maturation of EHC on quality of life and employment prospects should be considered. Visual-motor integration and visual-spatial integration have been found to be important measures that contribute to academic achievement (Carlson et al., 2013). Poorer handwriting is related to poorer visual-motor integration in normal children (Kaiser et al., 2009) and especially so in children with developmental coordination disorder (Wilmut et al., 2006; Bieber et al., 2016), attention deficit hyperactivity disorder (ADHD) (Stasik et al., 2009), autism spectrum disorder (Anzulewicz et al., 2016) and amblyopia (Engel-Yeger et al., 2012; Bieber et al., 2016), and thus emphasizes the need for a simple, objective measure of EHC.

Despite the development of good EHC by teen years, peak EHC does not continue through adulthood (Rand and Stelmach, 2011; Engel-Yeger et al., 2012; Ebaid et al., 2017; Low et al., 2017). In the current study, those over the age of 40 increasingly made more errors, took longer, and demonstrated greater variability in performance as the decades progressed. The deterioration in motor control during adulthood is well-known (Rand and Stelmach, 2011; Engel-Yeger et al., 2012; Ebaid et al., 2017; Low et al., 2017). Adults undertaking the Purdue Pegboard test (*n* = 7,834) showed a marked deterioration in dexterity with age (Tiffin and Asher, 1948), with similar outcomes on the more demanding Grooved Pegboard test (*n* = 357, 16–70 years) (Ruff, 1993). Superimposed upon aging itself, are other neural or physical factors in chronic conditions that are known to affect EHC such as arthritis (Suomi and Collier, 2003) or neurodegenerative diseases such as familial tremor (Trillenberg et al., 2006), Parkinson's disease (Boisseau et al., 2002), glaucoma (Kotecha et al., 2009), and Alzheimer's disease (Verheij et al., 2012). Clearly, acute conditions such as traumatic brain injury, including stroke, might also be expected to have profound effects on EHC (Gao et al., 2010; Rizzo et al., 2017), but the focus on functional assessment has to date been on motor coordination rather than sensory status or visuomotor integration (Ebaid et al., 2017; Low et al., 2017). On the other hand, one study assessing wrist-aiming found that older persons who are physically active do not appear to suffer as great a reduction in EHC performance as would be expected (Van Halewyck et al., 2014).

Regarding our choice of a tablet-device to assess EHC there are several considerations. First, one might question whether older generations may be unfamiliar using a stylus to trace on glass and thus confound results. Indeed, a number of our older participants did hold the stylus “in wonder” for a few moments and tentatively draw on the glass before commencing the practice trace. Any need for adaptation to proprioception or its impact on performance on the Slurp Test for participants unfamiliar with a tablet device was not pursued. However, the “castle” item we used as the practice item is itself a demanding trace requiring many changes in direction over a considerable distance (the average time for 101 participants was 28.3 ± 18.3 s). Hence it could well be that adaptation is achieved whilst tracing the castle. Furthermore, the fact that the 19-item pilot study (which included a number of older participants) used the Castle item as its practice item and showed no order effect across the subsequent 19 test items, is also suggestive that stylus-acclimatization is over before testing starts. Second, there may be limitations to the interpretation of the heterogeneity of outcomes due to the small number of participants in the 70+ age group (only seven participants using the iPencil® and seven a rubber-tipped stylus). However, an increasing heterogeneity in outcomes was already apparent in the next younger group aged 61–70 years (eight participants using the iPencil® and nine a rubber-tipped stylus), consolidating the notion that some older people are affected by age-related factors more so than others. A larger sample would facilitate elucidation of further factors that might contribute to these poorer performances.

Third, our choice to use a tablet device to test EHC in a two-dimensional space vs. the traditional three-dimensional reach and grasp/point style of EHC test, rests with the fact that we wanted to minimize upper arm involvement. This is important if one's test paradigm aims to assess subtle changes in the brain's integrity. As vision is such a widespread driver of human actions throughout the day (Bisley, 2011), in our opinion, detection of subtle changes in the brain requires assessment of fine motor control that is not contaminated by aberrations in the gross musculature. Children with developmental delay or some medical conditions and adults with medical/degenerative conditions affecting the shoulder and the arm may perform more poorly on reach and grasp or reach and point tasks than when undertaking simpler motor activities at a desk. Hence, our test commences once the subject has steadied themselves on the tablet. Thereafter, mainly fine motor movements of the wrist and fingers as driven by visual appraisal of the situation come under scrutiny.

The protocol for conducting the Slurp EHC Test warrants scrutiny due to its novelty. First, the optional sound alert was activated to indicate to the participant that they had deviated outside the straw. Having this alert present serves to pull the participant back into attending to the task, but one may ask whether it will detract from vision being the primary sense providing feedback to the visuomotor task at hand and thus introduce a ceiling effect and limit the magnitude of any deviation. Notably, when simply tracing along the straw there are no sound cues. We have preliminary data on the magnitude of the deviation which shows a similar age-related “starts poor, peaks, becomes poorer again” data set as do errors made and time (Junghans BM, et al. IOVS 2017;58:ARVO E-Abstract 5427). This implies that the sound cue announcing an error does not impose a ceiling on the magnitude of the deviation, although we cannot rule out a dampening effect. No studies have been undertaken to understand the impact of this when using the Slurp Test. Second, participants were to be limited to only 5 s to appraise the task ahead of them when their next item appeared. Although the investigator was never required to intercept, many participants could be seen to quickly survey the shape of the straw and smile upon realizing what shape they would trace next. Whether this scoping process creates a visuomotor map and enhances tracing outcomes has not been investigated. Importantly however, the benefit of this scoping phase with regards keeping the participant engaged with the test, perhaps in itself makes this brief instinctive limited scoping worthwhile. Third, the total time to undertake any EHC test is an important consideration. Whether a further reduction in the number of test items, to say just three, would yield the same EHC assessment outcomes is worth exploring.

For the researcher, the Slurp app offers new capabilities to quantify finer visuomotor performance in terms of space and time. At the end of testing, an option is available to download the database that yields the following information approximately every 0.1 s: x/y coordinates for the stylus, x/y coordinates of the midpoint of the straw nearest the stylus, the magnitude of the resultant deviation of the straw from the midpoint of the straw, categorically whether the deviation is/isn't deviating beyond the pre-set threshold, and the velocity of the stylus at each capture point. A pre-set error threshold of 3.5 mm was integrated by the app programmers to take pixilation effects into consideration. However, it is possible to change this sensitivity within the database retrospectively. A further benefit of the Slurp app is that the exact duration of each tracing task (some as short as 5–6 s) is captured digitally to a precision of 1000th of a second, which aligns with the importance of time as a sensitive measure of brain function (Miall and Reckess, 2002). The app's timing function is totally unobtrusive and thus assists the authenticity of the times captured.

In summary, digital eye-hand coordination testing offers a wide range of practitioners and scientists a level of understanding of the integrity of the brain that has hitherto been undertaken without sufficient attention or rigor in data capture. The Slurp (Lee-Ryan) Test app offers a quick and sensitive means of assessing the spatio-temporal aspects of eye-hand coordination in a manner acceptable to all age groups and applicable across a spectrum of situations in psychology, medicine and education.

## Ethics Statement

The study was approved by the University of New South Wales (UNSW) Human Research Ethics Advisory (HREA) Panel D: Biomedical. All participants were provided with a Participant Information Statement, and gave signed and informed consent after the study was explained in accordance with the Declaration of Helsinki. In the case of children who participated, one parent similarly signed consent, whereas the children themselves gave verbal consent.

## Author Contributions

BJ participated in conceptualization and design, research, collection of data, interpretation of results, drafting, and revision of the manuscript. SK participated in design, collection of data, data analysis, interpretation of results, and revision of the manuscript.

### Conflict of Interest Statement

The authors declare that the research was conducted in the absence of any commercial or financial relationships that could be construed as a potential conflict of interest. Whilst it might be perceived that the authors have the potential to gain financially from the sale of the Slurp app on iTunes, the funds go directly to the University of New South Wales with only a portion forwarded to the School of Optometry and Vision Science for the purpose of covering necessary upgrades and to maintain functioning of the app in response to upgrades to the Apple iOS.
